# Stress-induced cognitive dysfunction: hormone-neurotransmitter interactions in the prefrontal cortex

**DOI:** 10.3389/fnhum.2013.00123

**Published:** 2013-04-05

**Authors:** Rebecca M. Shansky, Jennifer Lipps

**Affiliations:** Laboratory of Neuroanatomy and Behavior, Department of Psychology, Northeastern UniversityBoston, MA, USA

**Keywords:** working memory, stress, catecholamines, glucocorticoids, sex differences, estrogen

## Abstract

The mechanisms and neural circuits that drive emotion and cognition are inextricably linked. Activation of the hypothalamic-pituitary-adrenal (HPA) axis as a result of stress or other causes of arousal initiates a flood of hormone and neurotransmitter release throughout the brain, affecting the way we think, decide, and behave. This review will focus on factors that influence the function of the prefrontal cortex (PFC), a brain region that governs higher-level cognitive processes and executive function. The PFC becomes markedly impaired by stress, producing measurable deficits in working memory. These deficits arise from the interaction of multiple neuromodulators, including glucocorticoids, catecholamines, and gonadal hormones; here we will discuss the non-human primate and rodent literature that has furthered our understanding of the circuitry, receptors, and signaling cascades responsible for stress-induced prefrontal dysfunction.

## Introduction

Our ability to manage, update, and act on information in the absence of external cues—executive functions collectively known as working memory—is critical to daily functioning (Arnsten and Castellanos, 2002). These processes depend on the structural and functional integrity of the prefrontal cortex (PFC) (Goldman-Rakic, 1996), a highly evolved brain region that guides emotion and behavior through projections to subcortical regions like the hypothalamus, amygdala, and brainstem nuclei (Price et al., 1996). Under optimal, stress-free conditions, microcircuits within the PFC work together to inhibit inappropriate responses and allow nuanced decision-making (Goldman-Rakic, 1995). Exposure to stress, however, can disrupt PFC function, markedly impairing working memory (Arnsten, 2009; Arnsten et al., 2012). From an ethological standpoint, this loss of complex processing may have once allowed more primitive behaviors to take precedence in order to aid survival. But today, non-life-threatening stressors can activate these same circuits, eliciting scattered thought, loss of focus, and judgment errors that can be detrimental to daily life, and—in extreme cases—lead to mental illness. Over the last few decades, animal research has helped elucidate the mechanisms that underlie these impairments, revealing a complex interaction between neurotransmitter signaling and hormone actions.

Working memory in animals is assessed using delay-based tasks, which require an animal to keep a piece of information in mind over the course of a delay period, in order to make an accurate choice when the delay ends. Monkeys performing the Delayed Response task must remember the location of a briefly presented stimulus on a screen, and then move their eyes to focus on that location. In rodents, the Delayed Alternation task requires the animal to remember which arm of a T-shaped maze it previously visited, and then visit the opposite arm on the subsequent trial. Both tasks involve dozens, or even hundreds of trials, and thus during the delay the animal must not only keep the “signal” (i.e., correct choice) in mind, but also suppress the “noise”—information from previous trials. Subsets of prefrontal neurons fire exclusively during the delay (Funahashi et al., 1989), suggesting a unique role for the PFC in this aspect of the task. Moreover, lesions of the PFC disrupt accuracy only when the task involves a delay (Funahashi et al., 1993), demonstrating that the PFC is not involved in the motor or motivational aspects of these tasks. Accurate performance on working memory tasks relies on the maintenance of a balanced neurochemical milieu in the PFC—one that is easily disrupted with exposure to stress.

Many kinds of mild stressors can impair working memory in animals. The most common stressor for monkeys is a loud white noise, which also disrupts working memory in humans (Arnsten and Goldman-Rakic, 1998). Stressors in rodents include brief restraint stress (Shansky et al., 2006), and administration of the anxiogenic drug FG-7142, a benzodiazepine inverse agonist (Shansky et al., 2004). Each of these manipulations activates the hypothalamic-pituitary-adrenal (HPA) axis, eliciting a cascade of hormone and neurotransmitter release that alters cognitive and emotional processes throughout the brain (Cordero et al., 2003; Mikkelsen et al., 2005). In this review, we will focus on the contributions of the catecholamines dopamine (DA) and norepinephrine (NE), and their interactions with glucocorticoids and estrogen.

## Dopamine and norepinephrine

The primary sources of DA and NE input to the PFC are the ventral tegmental area (VTA) and locus coeruleus (LC), respectively (Thierry et al., 1992). Selective lesions of these afferents impair working memory in monkeys, suggesting that baseline catecholamine signaling is required for optimal PFC function (Brozoski et al., 1979). Investigations into the downstream mechanisms by which these neurotransmitters mediate working memory—in both stress and non-stress conditions—indicate critical roles for the DA D1 receptor, and noradrenergic alpha-1 and alpha-2 receptors (Arnsten, 1998a).

**Figure 1:**
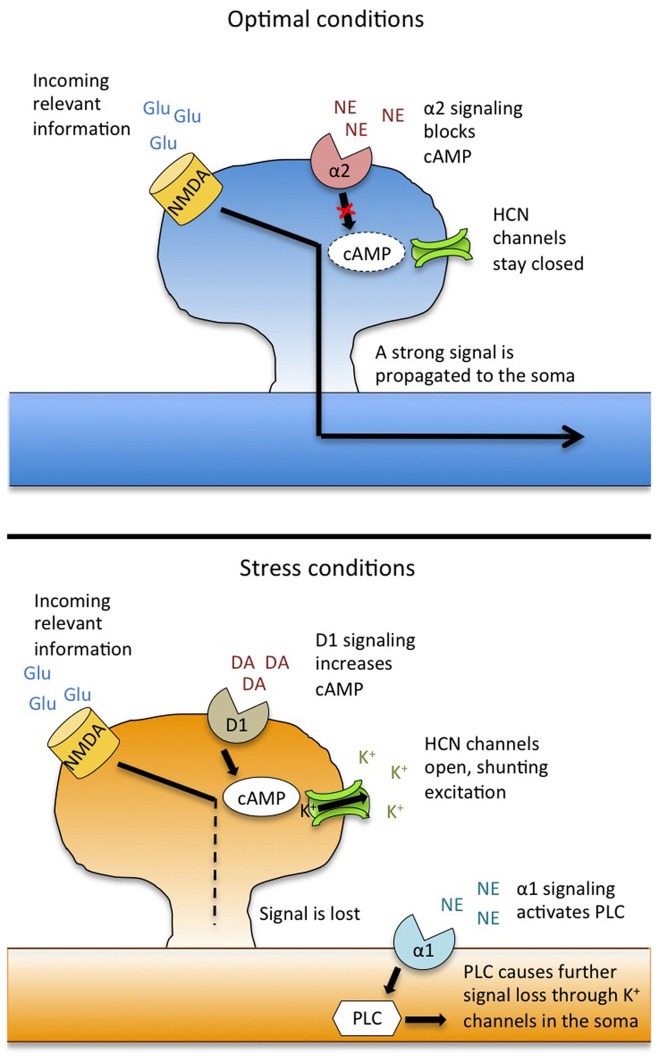
**Model for catecholamine modulation and stress-induced impairment of working memory.** Under stress-free conditions (**top**), the noradrenergic alpha-2 receptor drives activity in the prefrontal cortex by suppressing cAMP levels and strengthening the signal from incoming information. Under stress (**bottom**), overstimulation of the dopamine D1 receptor activates cAMP, causing HCN channels to open, resulting in a shunting of incoming excitation. Additionally, stimulation of NE alpha-1 receptors activates a PLC signaling cascade that causes further loss of excitation through K^+^ channels in the soma. This leads to a loss of information, and working memory failure. Adapted from Arnsten (2009) and Arnsten et al. (2012). Abbreviations: Glu, glutamate; NMDA, N-methyl D-aspartic acid receptors; NE, norepinephrine; DA, dopamine; HCN, hyperpolarization nucleotide-gated channels; PLC, phospholipase C.

The D1 receptor is coupled to the Gs protein, whose stimulation triggers a signaling cascade that involves increases in cyclic-AMP (cAMP) and protein kinase A (PKA), the effects of which are discussed below (Arnsten, 2011a,b). Pharmacological blockade of D1 receptors in both monkeys and rodents impairs performance on working memory tasks (Sawaguchi and Goldman-Rakic, 1991; Izquierdo et al., 1998), indicating a key role for D1 signaling in normal PFC function. Electron micrographs show that D1 receptors co-localize with glutamate receptors on dendritic spines (Pickel et al., 2006, and see Figure 1), making them strategically positioned to modulate incoming excitatory information. Single unit physiological studies in monkeys performing a delayed response task have revealed that D1 activity plays an integral role in filtering out “noise”—suppressing firing in PFC neurons that code for information irrelevant to the immediate task, thus increasing the likelihood of a correct response (Vijayraghavan et al., 2007). Without D1 stimulation, PFC neurons become generally overactive, rendering the animal vulnerable to distractions (Vijayraghavan et al., 2007).

While a lack of D1 activity can impair working memory performance, high levels of D1 stimulation also produce cognitive deficits—the classic “inverted-U” relationship. During stress, HPA axis activation leads to stimulation of the VTA, causing excess DA release into the PFC (Murphy et al., 1996). When this DA binds to the D1 receptor, its downstream signaling cascades lead to working memory impairment (Taylor et al., 1999). Accordingly, these impairments can be reversed by intra-PFC infusions of a D1 antagonist (Zahrt et al., 1997), as well as by infusions of cAMP and PKA inhibitors (Taylor et al., 1999). Physiologically, elevated D1 signaling leads to a suppression of not only “noise”-related neurons, but of “signal” neurons as well (Vijayraghavan et al., 2007)—the information is lost, and the PFC is unable to accurately guide behavior. Moreover, this general silencing of neuronal activity loosens the PFC's regulatory influence over subcortical structures, allowing amplified and protracted emotional responses (Arnsten, 1998b).

How does this switch take place on a cellular level? Recent work has revealed a critical role for hyperpolarization-activated/cyclic nucleotide-gated (HCN) ion channels, which co-localize on dendritic spines with D1 receptors (Paspalas et al., 2012). Traditionally, HCN channels serve to normalize neuronal membrane potential, opening to allow positive ions into the cell to combat post-firing hyperpolarization (Wahl-Schott and Biel, 2009). But as their name implies, HCN channels are also sensitive to changes in cAMP levels, and when cAMP increases (as happens when D1 receptors are over-activated), HCN channels open, letting Na^+^ and K^+^ flow *out* of the cell (Chen et al., 2007). The net effect of this efflux is a lessening of the likelihood that an incoming stimulus will be sufficiently excitatory to propagate an action potential, thus forming the physiological basis of D1-driven information loss. Pharmacological blockade of HCN channels restores working memory performance and PFC network tuning during stress or after administration of a D1 agonist, demonstrating a functional link between these channels and upstream changes in DA signaling (Arnsten, 2011b).

HCN channel activity is also modulated by the noradrenergic alpha-2 receptor. This receptor is coupled to Gi, the activation of which results in a decrease in cAMP. This causes a slowing of HCN channel conductance, thus preserving incoming excitatory input. In this way, the alpha-2 receptor acts to strengthen PFC network activity, enhancing the “signal” for relevant information, while as noted above, the D1 receptor suppresses “noise” (Wang et al., 2007). Thus, under optimal conditions, the D1 and alpha-2 receptors work together to fine-tune PFC neuronal firing. Pharmacological stimulation of the alpha-2 receptor can increase firing in PFC neurons that code for relevant information, enhancing working memory in monkeys and rodents (Wang et al., 2007). Additionally, alpha-2 agonists reverse working memory impairments that occur during stress (Birnbaum et al., 2000).

Alpha-2 receptors have a high affinity for NE, and are primarily bound and active during non-stress conditions (O'Rourke et al., 1994). Under stress, however, the LC releases NE throughout the brain and excess NE in the PFC binds instead to the lower-affinity alpha-1 receptor (Mohell et al., 1983). Stimulation of this receptor—either pharmacologically or because of stress-induced NE release—leads to working memory impairment and a silencing of PFC network activity (Arnsten et al., 1999). Conversely, administration of an alpha-1 antagonist can restore PFC function and neuronal firing during stress (Birnbaum et al., 1999). The impairing effects of alpha-1 stimulation are due in part to downstream activation of protein kinase C (PKC), the inhibition of which also reverses stress-related impairments on working memory tasks in monkeys and rodents (Birnbaum et al., 2004). The PKC pathway inhibits neuronal firing through the cleavage of membrane phoshoplipase C (PLC), which initiates phosphatidylinositol signaling (Birnbaum et al., 2004). Downstream, intracellular stores of Ca^2+^ travel to the soma and inhibit neuronal firing through opening of local K^+^ channels (Hagenston et al., 2008).

In summary, stress disrupts working memory by eliciting catecholamine release into the PFC, moving both DA and NE levels to the far end of their respective inverted U curves. Through DA D1 and NE alpha-1 receptor signaling, delay-related neuronal activity in the PFC is suppressed, and information critical to accurate task performance is lost (Figure 1). Because the PFC also helps to shut down the stress response, this loss of PFC function can lead to prolonged glucocorticoid release, which can exacerbate working memory impairments.

## Glucocorticoids

During emotional and stressful situations, activation of the HPA axis causes the adrenal cortex to release glucocorticoids, which travel through the bloodstream and cross the blood-brain barrier to activate glucocorticoid receptors (GRs) throughout the brain (De Kloet et al., 2005). While this release is critical to the enhancement of long term memories associated with the event (Rodrigues et al., 2009), glucocorticoid actions in the PFC impair working memory. Systemic injection of corticosterone in rats significantly reduces Delayed Alternation accuracy, and infusion of the GR agonist RU 28362 into the PFC similarly impairs working memory (Roozendaal et al., 2004). Finally, intra-PFC infusion of the GR antagonist RU 38486 reverses stress-induced impairments on the delayed spatial win-shift (DSWS) task, another test of prefrontal-dependent executive function (Butts et al., 2011). These findings suggest that glucocorticoids can impair PFC function through direct actions at GRs, but glucocorticoids may also indirectly exacerbate working memory impairments through interactions with the catecholamine systems described above.

One mechanism of interaction between glucocorticoids and catecholamines is the extraneuronal catecholamine transport system. These transporters are located on glia, and remove excess DA and NE from the synapse, helping to keep balanced and optimal stimulation of dopaminergic and noradrenergic receptors. Corticosterone blocks catecholamine transporters in the PFC (Gründemann et al., 1998), resulting in increased extracellular catecholamine levels. In this way, stress-induced glucocorticoid release in the PFC could lead to overstimulation of the both dopamine D1 and α1 noradrenergic receptors, thus producing PFC dysfunction.

Glucocorticoids also modulate dopaminergic transmission in the PFC. Dopaminergic cells in the VTA and PFC express GRs that become saturated during stress (Ahima and Harlan, 1990), altering the firing of dopaminergic projections. Interestingly, glucocorticoid effects on DA release in the PFC appear to be locally driven, rather than a result of actions in the VTA itself. *In vivo* microdialysis experiments show that an infusion of GR antagonist RU-38486 into the PFC suppresses stress-induced DA release, but infusions into the VTA have no effect (Butts et al., 2011). Therefore, GRs play a role specific to the PFC in modulating the magnitude of stress-induced DA efflux.

Finally, glucocorticoids may further exacerbate catecholamine effects by activating some of the same intracellular signaling pathways. As described above, α_1_ noradrenergic receptor stimulation during stress impairs PFC working memory through PKC intracellular signaling pathways (Birnbaum et al., 1999). Glucocorticoid release can also activate PKC signaling (ffrench-Mullen, 1995), thus potentially amplifying the effects of alpha-1 stimulation.

## Sex differences and estrogen effects

The vast majority of behavioral neuroscience research is conducted in male animals, and thus our general understanding of stress effects in the PFC is within the context of the male brain. From a translational standpoint, this is problematic; stress-related mental illnesses like post-traumatic stress disorder (PTSD) and major depressive disorder are twice as prevalent in women (Becker et al., 2007), suggesting a distinct neurobiology may underlie the stress response in female brains. Though the exact mechanisms have not yet been fully identified, a growing body of literature points to an important role for estrogen in modulating the neurotransmitter and glucocorticoid effects described above.

One of the first studies to investigate sex differences in stress-induced working memory impairments used the anxiogenic drug FG7142 to generate dose-response curves in male and female rats (Shansky et al., 2004). While T-maze performance declined with increasing doses in both sexes, females became impaired after lower doses of FG7142 than those required to impair males. When the authors divided the females based on estrus cycle phase, they found that this stress sensitivity was driven by females in proestrus, when estrogen levels are highest. Similar results were found after using increasing durations of restraint stress instead of FG7142 (Shansky et al., 2006), demonstrating generalizability of the effect, and providing evidence against a simple hormone-drug interaction.

Further support for the idea that high estrogen levels confer sensitivity to stress comes from studies in ovariectomized (OVX) female rats. OVX surgery removes circulating estrogen and progesterone, hormones that can be re-introduced via a subcutaneous time-release silastic capsule. After administration of low doses of FG7142, OVX rats with long-term estrogen replacement (OVX + E) demonstrate working memory impairments that are similar to those of females in proestrus, while OVX females with a blank capsule perform more like males—impaired only at higher doses (Shansky et al., 2009). In all of the above studies, high- and low-estrogen groups did not differ in baseline working memory performance, suggesting that estrogen does not directly mediate PFC function, but instead modulates the factors that contribute to stress-induced impairments. The mechanisms by which estrogen does this are not known, but several intriguing possibilities exist.

First, estrogen may exacerbate the effects of stress-induced glucocorticoid release. Female rats in proestrus have higher baseline serum corticosterone levels than males or females in diestrus, and females have a more robust corticosterone response to acute stress than males do (Mitsushima et al., 2003). Thus, females with high estrogen levels may be primed for an amplified corticosterone surge after exposure to lower levels of stress, eliciting working memory impairments through the mechanisms described above—either through direct actions at GRs, or through blockade of extraneuronal catecholamine transporters. To date, however, estrogen-glucocorticoid interactions have not been investigated in the context of stress-induced working memory impairments.

**Figure 2:**
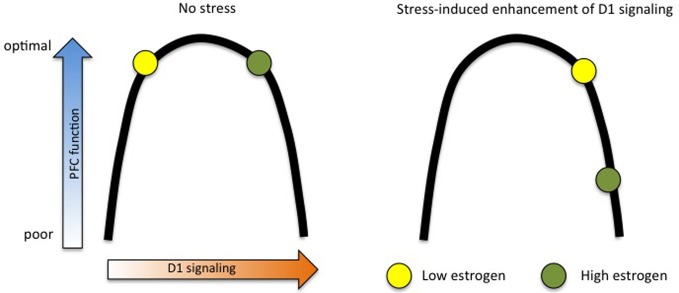
**Estrogen “ahead of the curve” hypothesis.** Estrogen may amplify the stress response in females by raising baseline dopamine D1 signaling, thus making small shifts more apparent in behavioral measures. In this model, high- and low-estrogen females perform equally well at working memory tasks under no-stress conditions, but mild stress shifts high-estrogen animals down into the far end of the D1 inverted U, while only pushing low-estrogen animals slightly across the middle.

Another means by which estrogen may sensitize the PFC to the detrimental effects of stress is through the dopaminergic system. Estrogen increases the physical number of dopaminergic projections from the VTA to the PFC (Kritzer and Creutz, 2008) and enhances extracellular DA concentrations (Xiao and Becker, 1994), putting it in a powerful position to modulate working memory. While these elevated DA levels may not have measurable behavioral outcomes on their own, they could indicate that high-estrogen females are “ahead of the curve” with respect to the D1-PFC function inverted U. In this scenario, mild stress merely pushes low-estrogen females just over the top of the U, while bumping high-estrogen females into impairment ranges. This hypothesis is illustrated in Figure 2.

The effects of elevated D1 signaling in high-estrogen females may be further exacerbated through estrogen's interactions with noradrenergic alpha-2a receptors. As described in the first section of this review, alpha-2a activity leads to decreased cAMP production and a closing of HCN channels, resulting in enhanced “signal” in PFC neurons coding for relevant information. This could serve to combat excess D1 activity, which leads to an opening of HCN channels, and a loss of information. Estrogen uncouples the alpha-2a receptor from its G-protein (Ansonoff and Etgen, 2001), thus potentially disrupting the delicate balance of D1 and alpha-2a activity that is required for optimal PFC function. In support of this idea, a dose of guanfacine (an alpha-2a agonist) that rescues stress-induced working memory impairments in males and OVX female rats has no effect in OVX rats with estrogen replacement (Shansky et al., 2009).

## Conclusions

Stressful events can lead to immediate and marked impairments in working memory, an executive function that depends on a balanced neurochemical state in the PFC. Research in non-human primates and rodents has shown that this impairment is driven by increased catecholamine signaling, which may be further modulated or exacerbated by changes in steroid hormone levels. Beyond stress, this work has provided critical insight into the mechanisms that underlie PFC function in general, and the potential for clinical application is substantial. Numerous mental illnesses—including Major Depressive Disorder, PTSD, Schizophrenia, and Attention Deficit/Hyperactivity Disorder [ADHD (Arnsten, 2007)]—are characterized by PFC dysfunction, and the pathways elucidated by the animal research described here are currently being targeted in pharmacological therapies. For example, the NE alpha-1 antagonist prazosin has been reported to be an effective treatment for PTSD (Berger et al., 2009), and the alpha-2 agonist guanfacine is used as an alternative to psychostimulant treatment for ADHD (Bidwell et al., 2011). Continued investigation into the neuromodulators that influence working memory—particularly in female populations—could lead to more nuanced and effective treatments for disorders that compromise prefrontal function.

### Conflict of interest statement

The authors declare that the research was conducted in the absence of any commercial or financial relationships that could be construed as a potential conflict of interest.
